# Synthetic condensate size correlates with yeast replicative cell age

**DOI:** 10.17912/micropub.biology.000582

**Published:** 2022-06-03

**Authors:** Meta Heidenreich, Joseph M Georgeson, Yotam Nadav, Emmanuel D Levy

**Affiliations:** 1 Weizmann Institute of Science

## Abstract

Yeast divides asymmetrically, with an aging mother cell and a ‘rejuvenated’ daughter cell, and serves as a model organism for studying aging. At the same time, determining the age of yeast cells is technically challenging, requiring complex experimental setups or genetic strategies. We developed a synthetic system composed of two interacting oligomers, which forms condensates in living yeast cells. Here, we report that these synthetic condensates' size correlates with yeast replicative age, making these condensates age reporters for this model organism.

**Figure 1. Synthetic protein condensate size correlates with yeast replicative cell age.**
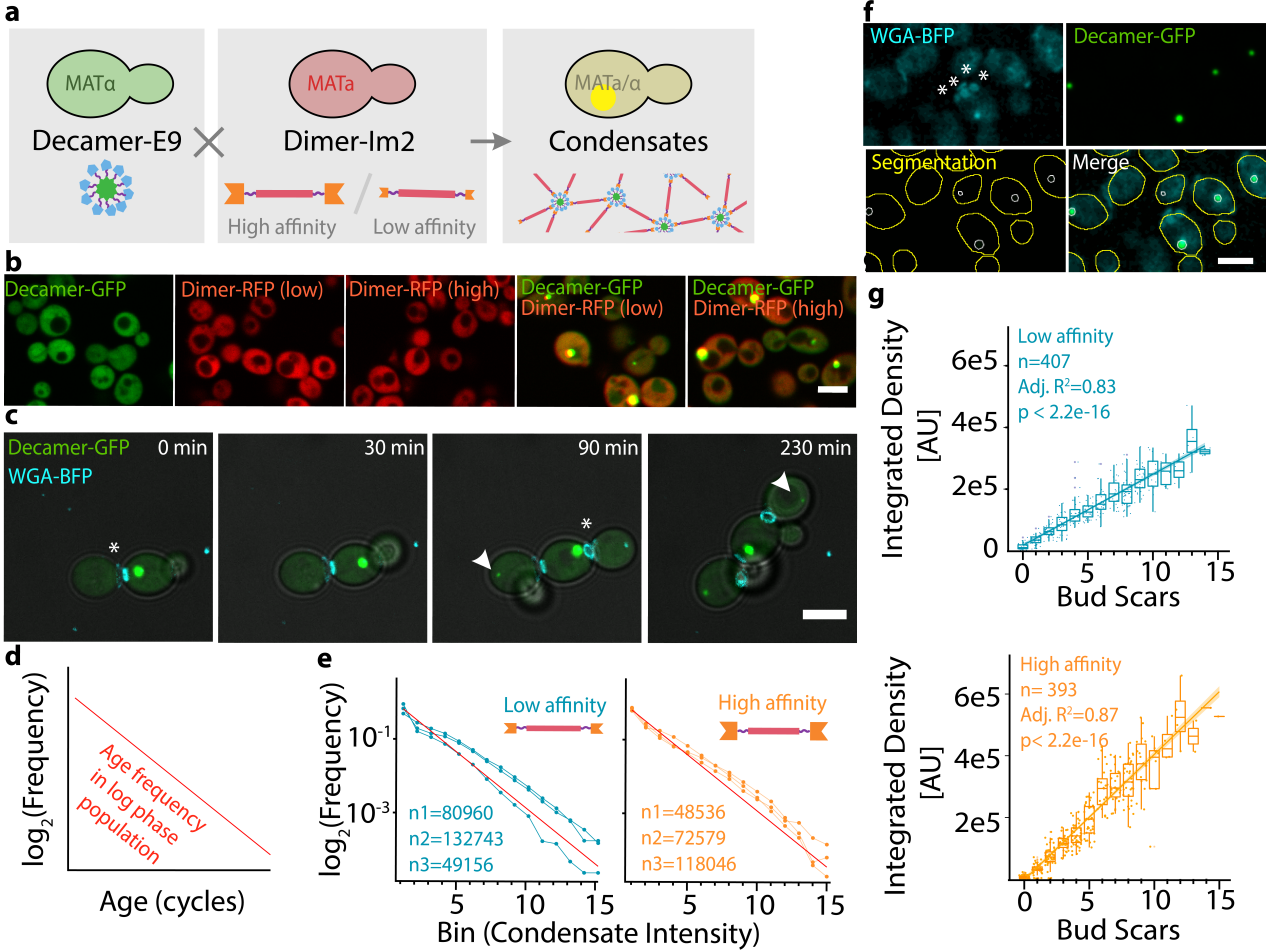
**a. Schematic of the synthetic condensate design.** The genetic construct encoding a previously described synthetic dimeric protein with interaction domains (Im2), with either high (wild type) or low affinity (E41A) for E9, was inserted into the genome of MATα mating type yeast. A cassette encoding a decameric protein fused to E9 was inserted into the genome of MATa type cells. The two resulting strains were mated and the diploid cells expressed both components forming synthetic mesh-like assemblies. The dimeric and decameric protein components were fused to red and green fluorescent proteins, respectively. **b. The assembly into condensates is dependent on the presence of both components.** Haploid yeast cells with only one component do not exhibit visible condensates, and the fluorescence signal is homogeneously distributed across the cytoplasm. Expression of both components after mating generates synthetic condensates. Diploid cells expressing dimer (RFP) together with the decamer (GFP) exhibit punctae, reflecting the formation of synthetic condensates. Components are indicated above. Yellow highlights the overlay of the red and green fluorescence signals. Scale bar = 5 μm. **c. Condensates appear within the first cell cycle of a newborn cell.** The appearance of a new budscar (cyan, white asterisks) is followed by the appearance of a condensate (green, white arrow) within 90 min. A timelapse series of growing cells expressing the synthetic condensates, co- stained for budscars is shown. Growing cells expressing the high-affinity dimer (not imaged) together with the decamer (GFP) were imaged in the presence of a bud scar stain. Timepoints are indicated above. Scale bar = 5 μm. **d. In a population of cells growing in log phase, the frequency of cells with a particular age is decreasing exponentially. e. Comparison of the theoretical age distribution to the distribution of condensate size.** Cells expressing the decameric component in combination with either the high affinity (orange) or low affinity dimer (cyan) were imaged, segmented, and the intensity within the condensate was recorded. Intensity measurements of condensates were binned to match the theoretically sampled replicative ages in the population. Individual lines indicate independent replicates of the experiment. The number of cells is given for each replicate, and the red line shows the theoretical frequency of cells of a particular age in the population. **f. Determination of condensate intensity and number of bud scars.** Bud scar staining (cyan) of cells expressing the high affinity dimer together with the decamer (green). Bud scars were counted manually. White asterisks highlight four bud scars on a cell. Cells and condensates were automatically segmented, and the condensate's GFP intensity was recorded. White and yellow lines indicate the identified cell and condensate boundaries, respectively. Scale bar = 5 μm. **g. Condensate size correlates with replicative cell age.** Bud scars were related to the condensate's integrated density in the GFP channel, as a measure of condensate size. Cells expressing the decamer with either the high-affinity dimer (orange), or the low-affinity dimer (blue), were used. Points indicate data for individual cells, boxplots show the distributions of condensate’s integrated densities for cells exhibiting a particular number of bud scars. The lower and upper hinges correspond to the first and third quartiles, the middle line indicates the median. The upper and lower whiskers extend to largest and smallest values, no further than 士1.5 * interquartile range. Solid lines show linear regressions of the integrated density of condensates versus the number of bud scars on the cells harboring them. Shaded areas show the 95% confidence interval of the linear model. Number of cells, adjusted R squared and p-values are indicated in the inset.

## Description

We recently developed a synthetic protein based system that forms condensates *in vivo* (Heidenreich et al. 2020) . The system consists of two proteins that interact and condense into a round, mesh-like structure. The structures grow over time, since the components are constitutively expressed. This hints that condensate size could be used as a molecular timer. Here, we show that such a system indeed serves as a good proxy for yeast's replicative age.

The previously developed version of the system was designed to yield heterogeneous expression of the two components in order to map phase diagrams. With such heterogeneous expression from plasmids, a majority of cells contain an excess of one component and do not form any condensate. Here however, our goal is that all cells contain a reporter of their replicative age, which requires the two components to be expressed in stoichiometric amounts. Therefore, we integrated the two constructs into two alleles of the same locus of diploid cells. Furthermore, to decrease the stoichiometry dependence of the formation of condensates, we increased the valency of one component from four to ten. Indeed, it was shown that increasing valency enhances phase separation of two-component systems over a wider concentration range (Nandi et al. 2019; Banani et al. 2016) . Finally, to assess the effect of affinity on the age-reporting reliability of our system, we utilized two different affinities between the interaction domains of the two components (Figure 1a).

As expected, condensates are only formed when both components are present, after mating two haploid strains where each haploid carries one component (Figure 1b). Timelapses of budscar-stained cells expressing our synthetic system revealed that condensates become detectable within the first cell-cycle of a newly born cell (Figure 1c, Movie 1).

In a dividing cell population, the frequency f _A_ of cells of age A is f _A_ = 1 / 2 ^A^ , so that half of the population has not yet divided (age 1), a quarter of the population is one cycle old (age 2) and so on (Figure 1d). To examine whether the condensate’s size recapitulates such an age-distribution, we measured the condensate sizes across a large number of cells (N), binned the results into log _2_ (N) bins, and compared it to the theoretically expected distribution of ages. We observed that the size-distribution of condensates in the population matched the expected distribution of ages, and the higher affinity version of the system appeared more accurate (Figure 1e). To further validate that condensate size serves as a good proxy for cell age, we stained and manually counted budscars of cells expressing our synthetic system, and correlated this number to the size of automatically identified condensates (Figure 1f). The number of budscars, and therefore yeast replicative age, correlated well with condensate size (R ^2^ =0.83 and 0.87 for the low- and high affinity versions of our system, respectively, Figure 1g). Interestingly, such correlation implies that these synthetic condensates are rarely passed to daughter cells, presumably due to their size prohibiting passage through the bud neck.

The identification or isolation of old cells by micro-dissection is laborious. Alternative methods, such as microfluidic techniques (Jo et al. 2015; Lee et al. 2012; Chen, Crane, and Kaeberlein 2017; Crane et al. 2014) , the mother enrichment program (Lindstrom and Gottschling 2009) , or a magnetic-bead based enrichment of aged cells (Hendrickson et al. 2018) are difficult to upscale for genome-wide studies. The approach presented here, where a protein’s assembly serves as a reporter for age, could be a powerful alternative owing to the ease with which age can be inferred by this method. More generally, synthetic assemblies in cells find unexpected applications, such as the use of synthetic protein filaments (Garcia-Seisdedos et al. 2017) as ticker-tape recorders of intra-cellular events (Linghu et al. 2021; Lin et al. 2021) . It is our hope that this system will serve the community in novel applications.

## Methods

The components were adapted from our previously developed system (Heidenreich et al. 2020) . We used the dimer fused to FusionRed and Im2 with either wild type (1.5 ± 0.1 x 10 ^-8^ M), or low affinity (E41A) for E9: 3*10 ^-5^ M (Li et al. 1998) , in combination with a decameric oligomerization domain (1VPX (Joint Center for Structural Genomics (JCSG) 2004) ) fused to E9 and Venus. To generate homogeneous expression, both components were driven by the same promoter (TDH3 promoter) and terminator (CYC terminator). The constructs were cloned into m3925 plasmids (Voth, Jiang, and Stillman 2003) that were modified to carry G418 (decamer) or hygromycin (dimer) resistance. Finally, the constructs were inserted into the TRP1 locus of of yeast strains of opposite mating type, where the dimer was inserted into MATα type (BY4741), and the decamer into MATa type (BY4742) cells (Brachmann et al. 1998) . The two resulting strains were mated, yielding diploid cells (BY4743) carrying both components (Figure 1 a and b). The plasmid and strain details are listed in Table 1 (Reagents).

Cells were grown in synthetic defined (SD) media with hygromycin and G418 to logarithmic growth (OD _600_ 0.6-0.8) overnight. For staining budscars, cells were transferred to optical 96-well plates (Greiner ^TM^ ) and WGA-CF405M (Biotium) was added to a final concentration of 50 μg/ml 20-30 min prior to imaging. Images were acquired using an Olympus IX83 microscope with a Yokogawa CSU-W1 spinning disc confocal scanner and an automated piezo-stage (Mad City Labs). For the time-lapse movie of budscar stained cells (Figure 1c), seven z-stacks were acquired and maximum intensity z-projections were generated using FIJI (Schindelin et al. 2012) . For analyzing the condensate’s integrated intensity, 100 images with eight z-stacks were acquired and an average z-projection was used (Figure 1e-h). Cells and condensates were segmented, and the condensate’s intensity was recorded using custom scripts (Matalon et al. 2018) . To correlate the condensate’s integrated intensity to the number of budscars, cells and condensates were segmented automatically. Then, tens of cells with each number of budscars were identified manually and the preceding segmentation was used to measure the corresponding condensate’s integrated intensity. Data analysis and plotting was conducted using custom R scripts.

## Reagents

Table 1: Plasmids and strains used and generated in this study.

**Table:** 

Plasmids			
Name	Backbone Plasmid	Description	Comment
pMH14 (Heidenreich et al. 2020)	m3925	GPDpromoter-NES-Im2-4LTB-FusionRed-CYCterminator-HygromycinR	High affinity dimer integration plasmid for TRP1 locus
pMH16	m3925	GPD promoter- Venus-1VPX-E9-CYCterminator-KanMX	Decamer integration plasmid for TRP1 locus
pMH18	m3925	GPDpromoter-NES-Im2(E41A)-4LTB-FusionRed-CYCterminator-HygromycinR	Low affinity dimer integration plasmid for TRP1 locus
Strains			
Name	Background Strain	Genotype	Description
yMH14	BY4742	MATα his3Δ1 leu2Δ0 lys2Δ0 ura3Δ0 trp1::GPDpromoter-Venus-1VPX-E9-CYCterminator-KanMX	MATα with decamer in TRP1 locus, KanMX resistant
yMH15	BY4741	MATa his3Δ1 leu2Δ0 met15Δ0 ura3Δ0 trp1::GPDpromoter-NES-Im2-4LTB-FusionRed-CYCterminator-HygromycinR	MATa with high affinity dimer in TRP1 locus
yMH16	BY4741	MATa his3Δ1 leu2Δ0 met15Δ0 ura3Δ0 trp1::GPDpromoter-NES-Im2(E41A)-4LTB-FusionRed-CYCterminator-HygromycinR	MATa with low affinity dimer in TRP1 locus, Hygromycin resistant
yMH17	BY4743	MATa/alpha; his3D1/his3D1; leu2D0/leu2D0; met15D0/MET15; LYS2/lys2D0; ura3D0/ura3D0 trp1/trp1::GPDpromoter-Venus-1VPX-E9-CYCterminator-KanMX/GPDpromoter-NES-Im2-4LTB-FusionRed-CYCterminator-HygromycinR	MATa/α diploids with high affinity dimer and decamer in the two TRP1 alleles, KanMX and hygromycin resistant, generated by mating yMH14 and yMH15
yMH18	BY4743	MATa/alpha; his3D1/his3D1; leu2D0/leu2D0; met15D0/MET15; LYS2/lys2D0; ura3D0/ura3D0 trp1/trp1::GPDpromoter-Venus-1VPX-E9-CYCterminator-KanMX/GPDpromoter-NES-Im2(E41A)-4LTB-FusionRed-CYCterminator-HygromycinR	MATa/α diploids with low affinity dimer and decamer in the two TRP1 alleles, KanMX and hygromycin resistant, Generated by mating yMH14 and yMH16
